# Bio-Based Polybenzoxazine–Cellulose Grafted Films: Material Fabrication and Properties

**DOI:** 10.3390/polym15040849

**Published:** 2023-02-08

**Authors:** Thirukumaran Periyasamy, Shakila Parveen Asrafali, Seong-Cheol Kim

**Affiliations:** School of Chemical Engineering, Yeungnam University, Gyeongsan 38541, Republic of Korea

**Keywords:** amino cellulose, curcumin, stearylamine, benzoxazine, bio-based films

## Abstract

Despite the fact that amino cellulose (AC) is biodegradable, biocompatible, and has excellent film-forming properties, AC films have poor mechanical properties and are not thermally stable. An AC-based composite film prepared from AC and curcumin-stearylamine based benzoxazine (C-st) is reported in order to improve its performance and promote its application. As starting materials, C-st and AC were used to produce a C-st/AC composite film possessing a synergistic property through chemical cross-linking and hydrogen bonds. Two salient features with respect to the curing behavior were obtained. Firstly, the onset of curing was reduced to 163 °C when the benzoxazine monomer was synthesized from fully bio-based precursors (such as curcumin and stearylamine). Secondly, a synergistic effect in curing behavior was obtained by mixing C-st with AC. As a result of tensile tests and thermal analysis, the poly(C-st) benefited the composite films with pronounced mechanical and thermal properties, even at elevated temperatures. There was a 2.5-fold increase in tensile strength compared to the AC film, indicating that the composite films have the potential to be used for functional purposes. These poly(C-st)/AC films with improved mechanical and thermal properties have the ability to replace naturally occurring polymer films in film-related applications.

## 1. Introduction

There is a growing demand for petroleum-derived plastics in the modern world. This would obviously lead to the polluting of the environment to a greater extent. Hence, these days, a lot of effort is focused on replacing these petroleum-derived polymers with sustainable, biodegradable, and environmentally friendly polymers. Nevertheless, these types of polymers generally come with a number of drawbacks with regard to their mechanical and thermal performance. Alternatively, such disadvantages could be overcome by blending these biopolymers with synthetic materials. It is possible to improve the properties of these polymers by copolymerization [1,2,3,4,5]. The hybridization of bio-polymer with synthetic polymer may result in a polymerized product with improved properties with a wide range of applications. For several years, significant interest has been raised in these two natural polymers, viz., chitosan and cellulose. Among their unique properties, which include biocompatibility and biodegradability, a major factor determining their satisfying application is their non-toxic nature [6,7,8,9,10].

A plant cell wall is made of cellulose, a biopolymer that is abundant and widely distributed. By chemical reactions, cellulose can be converted into its derivatives, thus converting its functional applications. The presence of many reactive hydroxyl groups in cellulose can form a greater number of cellulose derivatives through chemical reactions. A transparent film with substantial mechanical strength can be formed using cellulose and its derivatives due to their excellent water absorption properties [11,12,13,14]. A natural process for achieving this is through the reaction of amine compounds with cellulose to produce amino cellulose (AC) with terminal -NH_2_ groups. AC forms good films as it is soluble in water and acts as a good cross-linker in film-forming applications because of its hydrophilic –OH and –NH_2_ groups. AC films can be produced directly, but their low thermal and mechanical properties limit their applications. It is possible to modify or disable the inherent disadvantages of natural polymers by combining them with synthetic polymers to improve their properties. Therefore, to enhance the properties of biofilms, it is necessary to use a high-performance synthetic polymer [15,16,17,18].

As one of the most attractive phenolic resins on the market, polybenzoxazine (PBz) has excellent mechanical, physical, and thermal properties as well as molecular design flexibility. Benzoxazine can be synthesized by Mannich condensation using inexpensive raw materials. Furthermore, they do not produce any by-products during polymerization and are cured without using a catalyst [3]. The linearization or cross-linking of benzoxazine monomers is determined by the heterocyclic ring opening. In addition to having excellent dimensional stability and excellent processability, polybenzoxazine has a broad range of molecular design flexibilities [6,7]. Despite the fact that polymers are very strong and despite all these advantages, their application is limited by their brittle nature. A material’s toughness can be improved [8,9,10] in order to take advantage of PBz’s potential advantages. Two approaches have been found to be effective for improving the performance of polybenzoxazine: first, novel benzoxazines can be synthesized by altering their structure; second, composites of polymers and inorganic fillers may be produced to enhance the performance of polybenzoxazine [19,20,21]. Benzoxazine monomers undergo ring-opening polymerization without catalysts or initiators during the polymerization process, with no toxic by-products or volatile compounds generated. Research has been focused on benzoxazine polymers in recent years due to their thermoplastic properties such as ductility, processability, and film formation in combination with their thermosetting properties such as chemical resistance and dimensional stability. Though there are many appreciable properties, the brittleness of neat Pbz is still a problem to overcome, one which could be rectified by preparing Pbz-based blends, alloys, and composites with epoxy, polyurethane, polystyrene, poly(methyl methacrylate), and polycarbonate. In this way, Pbz blends/alloys/composites with improved properties could be obtained [22,23,24,25,26,27,28,29,30].

Curcumin, chemically known as 1,7-bis-(4-hydroxy-3-methoxyphenyl)-1,6- heptadiene-3,5-dione, is a yellow polyphenolic compound found in turmeric that comes under the family of rhizomes. Curcumin has potential pharmacological activity and excellent therapeutic efficacy. Furthermore, curcumin’s functional phenolic compound enhances the performance of the polymer containing the curcumin moiety. To the best of our knowledge, curcumin has rarely been used to synthesize benzoxazine. Resins containing curcumin should have greater sustainability, antibacterial, antiviral, chemical, and physical resistance properties when incorporated into benzoxazine [31,32,33,34,35].

In recent years, renewable organic materials have been used to develop benzoxazines. Additionally, some benzoxazine monomers have been reported to be soluble in water and aqueous solutions, making them environmentally friendly. Hence, in this study we made an effort to incorporate the curcumin moiety in the benzoxazine structure (C-st) and to blend it with AC to fabricate poly(C-st)/AC films. Hopefully, the synergistic effect of all the components and the processes, including Mannich condensation, ring-opening polymerization, and cross-linking, can be utilized to produce films with improved properties of their own.

## 2. Materials

Cellulose (microcrystalline powder, 20 µm), curcumin, stearylamine, and paraformaldehyde were purchased from Sigma-Aldrich (Steinheim, Germany). Dimethyl sulfoxide (DMSO) N, N-dimethylacetamide (DMAc), anhydrous LiCl, acetic acid, sodium hydroxide (NaOH), triethylamine, and p-toluenesulfonyl chloride were supplied by Duksan Chemicals Co., Ltd., Ansan, Republic of Korea. Ethanol was supplied by Daejung Chemical Company, Ltd., Siheung, Republic of Korea. All the chemicals were used without further purification.

### 2.1. Instrumentation Methods

The synthesized material was thoroughly characterized by various physicochemical techniques. The Fourier transform infrared (FT-IR) spectrum was obtained with a Perkin Elmer MB3000 FTIR spectrometer. The spectrum was obtained at a resolution of 4 cm^−1^ in the IR range of 400–4000 cm^−1^. The samples were prepared by grinding them with KBr and compressing them to form discs. The nuclear magnetic resonance (NMR) spectrum was recorded with an Agilent NMR, VNS600 at a proton frequency of 600 MHz for ^1^H-NMR. For the differential scanning calorimetric (DSC) analysis, five to ten milligrams of the sample was placed in the sample pan, crimped, and heated to 10 °C min^−1^ in an atmosphere of N_2_ using the TA instrument Q10 model. The Instron E300LT, Universal Tester was used to evaluate the mechanical properties of the cross-linked films under uniaxial tension. A total of five specimens of each composition were tested at ambient temperature using a cross-head speed of 1 mm/min, and the average value was determined in order to calculate the modulus values. We used a thermal analyzer TA Q600 to analyze the thermal stability of the films. The cured samples were heated in an open silicon pan up to 800 °C in a N_2_ atmosphere at a heating rate of 20 °C min^−1^. The field emission scanning electron microscopic (FESEM) images were obtained with an accelerating voltage of 4 kV using an S-4800 equipped with a 4 kV accelerating voltage.

### 2.2. Synthesis of Amino Cellulose (AC)

According to the method published in the literature by Heinze, the AC was prepared as described below following a three-step procedure. To begin with, 5.0 g of cellulose was dissolved with a N, N-dimethylacetamide (DMAc)/anhydrous LiCl mixture. The homogeneous cellulose solution that was suspended in DMAc (15 mL) was made into a base agent by adding a solution of tosyl chloride (11.9 g) to it dropwise. The reaction proceeded at 8 °C for 24 h, and the resultant mixture was in ice water. The product was washed several times with deionized water and ethanol and finally dried under a vacuum at 50 °C to obtain p-tosyl cellulose (TC). The final step involved adding tetraethylenepentamine (26.5 g) to the TC (2 g) that contained the DMSO solution and mixing for 6 h at 100 °C. Following the precipitation of the mixtures in acetone, the mixtures were washed with acetone and ethanol, followed by drying under a vacuum at 50 °C to obtain amino cellulose (AC). The yield was 84%.

### 2.3. Synthesis of Curcumin-Stearylamine-Based Benzoxazine (C-st)

**Scheme 1:**
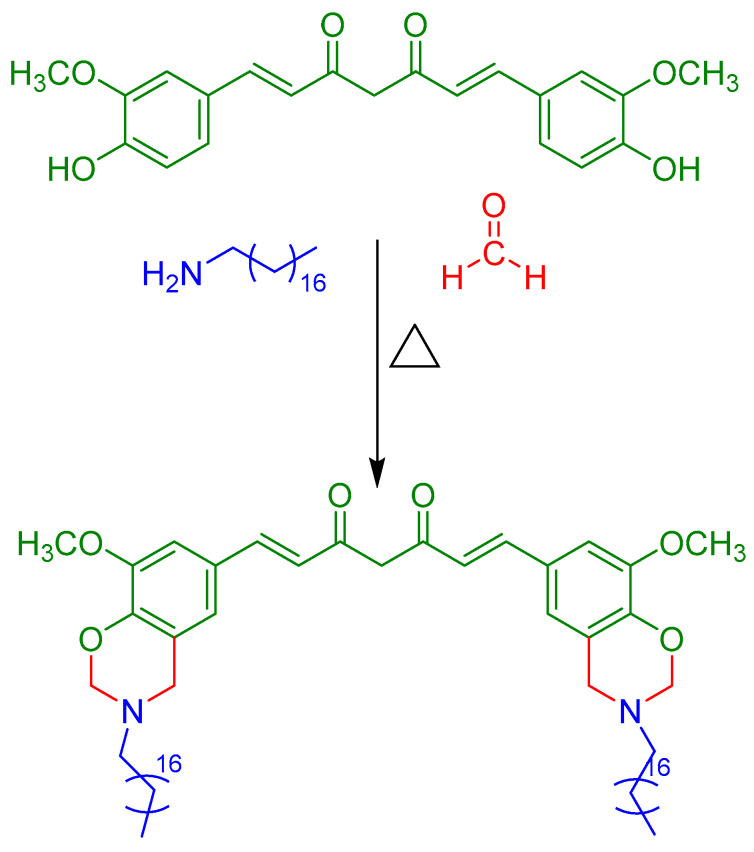
Synthesis of benzoxazine monomer, C-st.

In a 250 mL round-bottomed flask, 80 mL of anhydrous chloroform was added to 3.68 g of curcumin (0.01 mol), 1.20 g of paraformaldehyde, 5.39 g of stearylamine, and 0.25 g of CaH_2_. The reactants were kept for stirring and refluxed at 120 °C for 8 h. After the completion of the reaction, the reaction mixture was cooled to room temperature and filtered to obtain the liquid, which was then evaporated under reduced pressure to remove the redundant solvent. An excess of methanol was gradually added to the residue solution, and the precipitate was washed several times with deionized water before being dried under a vacuum at 60 °C for 24 h (yield: 85%) (Scheme 1).

### 2.4. Fabrication of Poly(C-st) and Poly(C-st)/AC Composite Films

For the preparation of pristine poly(C-st) biofilms, 5 g of curcumin-stearylamine-based benzoxazine (C-st) was dissolved in 50 mL of 1% acetic acid. After agitating the contents for one hour, a homogeneous mixture was formed. A uniform thickness of biofilm (1 mm) was obtained by pouring this homogeneous solution into a Petri dish (80 mm diameter) and drying at room temperature for 24 h. A homogeneous solution weighing 40 g was needed to produce 1 mm thick films after several trials. Another 24 h at 50 °C was spent drying the cast film in a vacuum oven. The film was sequentially heated for one hour at 100, 150, 200, and 250 °C to complete the final cross-linking of poly(C-st). Various amounts of poly(C-st) and AC were combined to form the composite films. Each composition consisted of 100 mL of acetic acid aqueous solution, which was the same amount as the combined precursors. After dissolving AC in 100 mL of acetic acid, C-st was added to create a homogeneous solution. As before, casting was performed to produce the films. The weight ratios of the C-st and AC are listed in Table 1.

## 3. Results and Discussion

### 3.1. Structural Characterization of Amino Cellulose (AC)

The amino cellulose synthesis is a result of a nucleophilic substitution reaction, in which the hydroxyl groups of cellulose are replaced by amine groups through the formation of tosyl cellulose as an intermediate. The FT-IR spectrum was used to determine the chemical structure of AC. The chemical compositions of cellulose, tosyl cellulose, and amino cellulose are visible in the FT-IR spectrum. As a result of the cellulose absorption, these peaks are typically related to the stretching or bending vibrations of –OH, –CH, absorbed water, –CH_2_, C–O–C, and β-form glycosidic bonds at 3330, 1584, 1413, 1073, and 891 cm^−1^, respectively. The tosyl cellulose had some distinctive peaks that were attributed to the p-substituted benzene ring (1589, 1500, 1480, and 802 cm^−1^) and O=S=O group (1371 and 1168 cm^−1^). The amino cellulose spectrum shows new absorption peaks corresponding to the C–N, -NH_2_, and –NH groups. Furthermore, the spectrum shows a peak at 1152 cm^−1^, which represents C−N−C symmetric stretching vibrations. The characteristic peak appearance at 1597 cm^−1^ is due to the amino group of tetraethylenepentamine. Additionally, the methylene groups (−CH_2_) in tetraethylenepentamine caused asymmetric and symmetric stretching vibrations in the range of 2914 to 2827 cm^−1^.

### 3.2. Structural Characterization of Benzoxazine Monomer (C-st)

**Figure 1:**
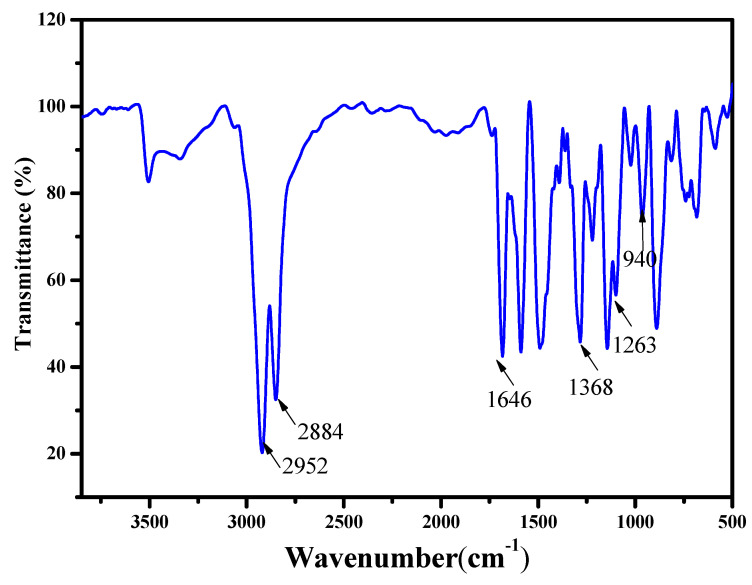
FT-IR spectrum of C-st.

**Figure 2:**
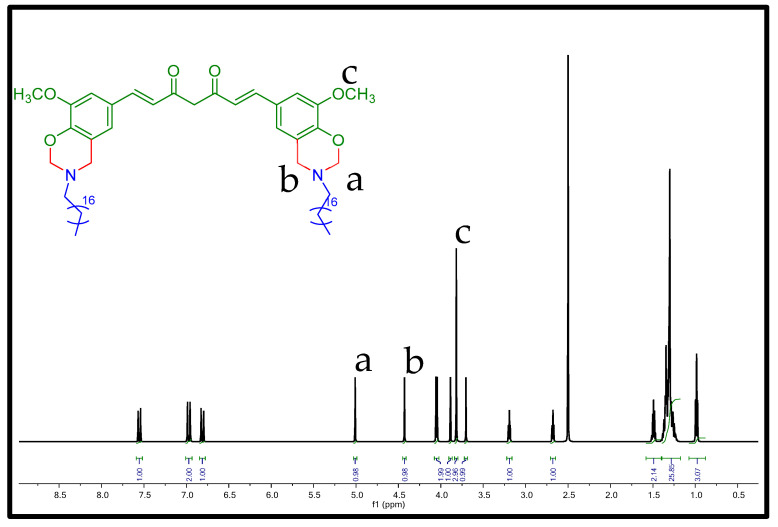
^1^ H-NMR spectrum of C-st.

The FT-IR spectrum of the synthesized C-st benzoxazine precursor is shown in Figure 1. The asymmetric and symmetric stretching vibrations of the C-O-C bond produce the absorption peak at 1263 and 1255 cm^−1^, and the benzoxazine ring structure produces the absorption peak at 940 cm^−1^. The absorbance at 1646 cm^−1^ is attributed to the –C=O stretching vibrations of the amide group in chitosan. The asymmetric and symmetric C-H stretching vibrations of the benzene rings and stearylamine are observed at 2952 and 2884 cm^−1^, respectively [36,37,38]. Additionally, there is a band at 1368 cm^−1^ due to the tetra substituted benzene ring. Figure 2 represents the ^1^H-NMR spectrum of C-st. The signals at 4.6 and 5.4 ppm represent the classic protons associated with the oxazine ring, Ar–CH_2_–N, and O–CH_2_–N, respectively. Between 1 and 1.5 ppm, a hydrogen signal is detected due to the methyl moiety of the long aliphatic chain in stearylamine. The singlet at 3.75 ppm is due to the –OCH_3_ protons from the curcumin moiety. Allyl protons are associated with doublets at 2.6 and 3.2 ppm. A multiplet between 6.3 and 7.0 ppm is assigned to the aromatic protons. The above results indicate the formation of the C-st benzoxazine precursor [39,40,41].

### 3.3. Polymerization Behavior of C-st/AC Blends

**Scheme 2:**
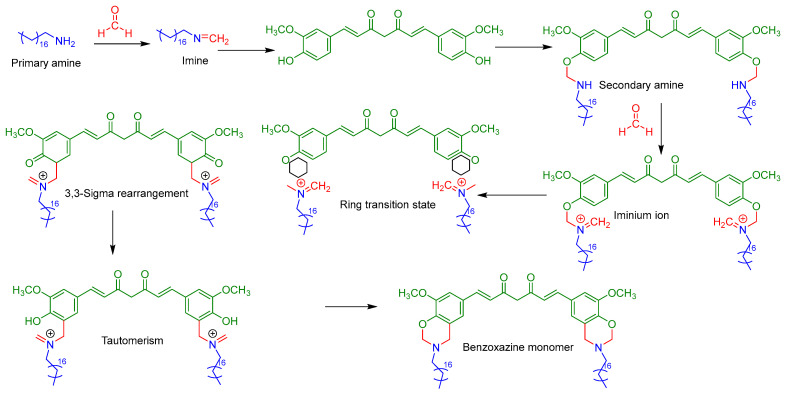
Mechanism of formation of benzoxazine monomer.

**Scheme 3:**
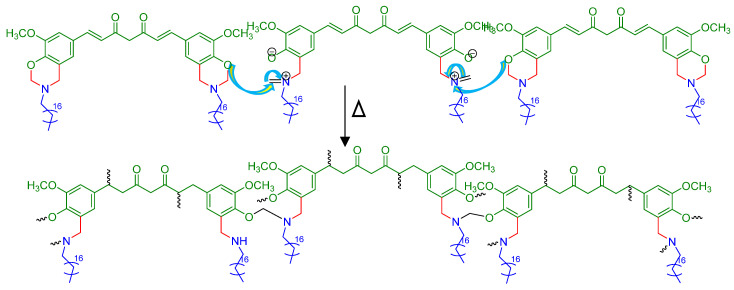
Mechanism of ring-opening polymerization.

**Figure 3:**
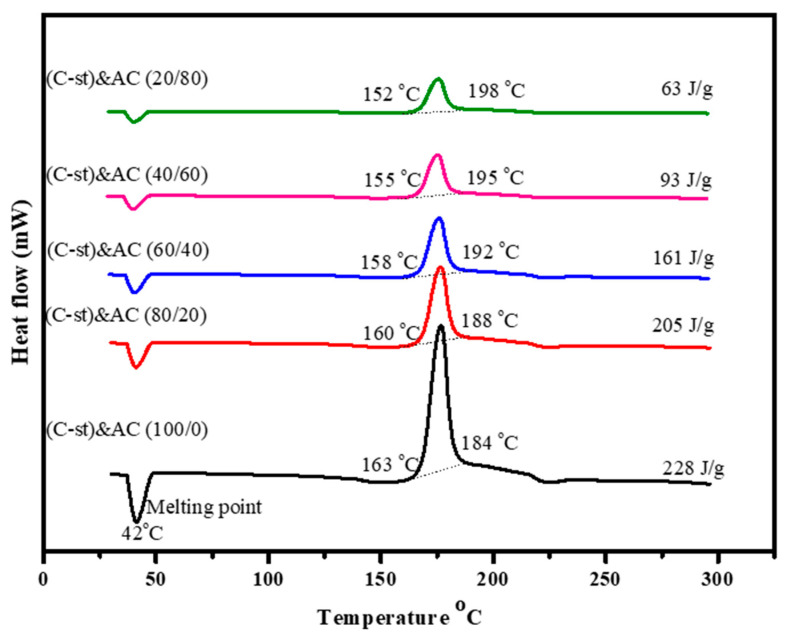
DSC thermograms of C-st/AC blends.

As shown in Scheme 2, C-st is derived from curcumin, stearylamine, and paraformaldehyde via Mannich condensation, forming the oxazine ring. According to Scheme 3, the C-st polymerization occurred via thermally mediated ring-opening polymerization. By cleaving O–CH_2_–N within the oxazine ring, zwitterionic intermediates are formed with the cationic moieties. Electrophilic substitution occurred between the adjacent benzoxazine monomers and the cationic imine moieties. Thus, a new type of Mannich bridge was formed by rearranging the structure. Using DSC, the benzoxazine polymerization behavior was also monitored with different C-st/AC weight ratios. The C-st/AC blends showed entirely lower exothermic peaks than the neat benzoxazine, as shown in Figure 3, whose data are tabulated in Table 2. With the high AC content (about 80%), the onset and maximum curing temperatures dropped from 163 and 175 °C to 152 and 170 °C, respectively. Benzoxazine opens up more readily when it comes in contact with reactive amine groups, which promotes ring-opening polymerization. In a similar way, Yang and Gu [42] reported similar curing behavior for a benzoxazine monomer containing benzimidazole groups. The benzimidazole group plays the role of a catalyst that could be recognized within the network of the compound. This benzimidazole group being in part of the network structure helps in the protonating of the oxygen atoms in the oxazine rings to form iminium ions. In general terms, the lowering of the polymerization temperature of the benzoxazine precursor is necessary in order to avoid the partial degradation of the components. In contrast to the simple catalytic effects of amine groups, AC affects the polymerization of benzoxazine in a more complex manner. The polybenzoxazine formed with hydrogen bonds exhibits a higher glass transition temperature than a polybenzoxazine that is not polymerized with such compounds. As a result, hydrogen bonding species may interact synergistically, with interfering effects on propagation, to produce the polymer. An intramolecular hydrogen bond is probably responsible for the occurrence of this phenomenon between the nitrogen atom of the Mannich base and the phenolic group within the molecule [28,43,43,44]. Multifunctional groups should be studied in more detail before being understood as factors in catalysis and hydrogen bonding. According to the DSC thermograms, the polymerization enthalpy decreased from 228 to 63 J/g as the AC increased. Moreover, the polymerization enthalpy decreased as a consequence of the decreased benzoxazine monomer (C-st) content in the mixture (C-st/AC). Furthermore, polybenzoxazines contain the phenolic hydroxyl group (–OH) and electronegative atoms (such as oxygen and nitrogen) in their structure, and therefore, strong intra- and intermolecular hydrogen bonding exists between them. This unique combination of intra- and intermolecular hydrogen bonding contributes to the improvement in the properties of the polybenzoxazines. Moreover, AC contains hydroxyl (–OH) and amine (–NH_2_) groups in its structure. Moreover, these groups in AC can also make additional contributions to the hydrogen bonding interactions between AC and Pbz. In the present work, the solubility of the benzoxazine in aqueous solution offers a versatile pathway to a unique hydrogen bonding capability. In addition to this, the presence of secondary amine (−NH) in the structure of AC also contributes to hydrogen bonding. Furthermore, during the ring-opening polymerization, the benzoxazine ring opens up, producing –OH groups and short side groups between each molecule. These –OH and side groups interact with the –OH and –NH_2_ groups in AC through hydrogen bonding interactions. The mechanism of these hydrogen bonding interactions between the –OH and –NH of benzoxazine was investigated previously. Hence, it is clearly evident that the –OH, –NH_2_, and –NH groups of Pbz and AC are responsible for these interactions. These strong intra- and intermolecular hydrogen bonding interactions between the polymer chains bring out the synergistic effect of both the Pbz and the AC, producing high-performance polymer blends.

### 3.4. Phase Morphology of Poly(C-st), AC, and Poly(C-st)/AC Composite Films

**Scheme 4:**
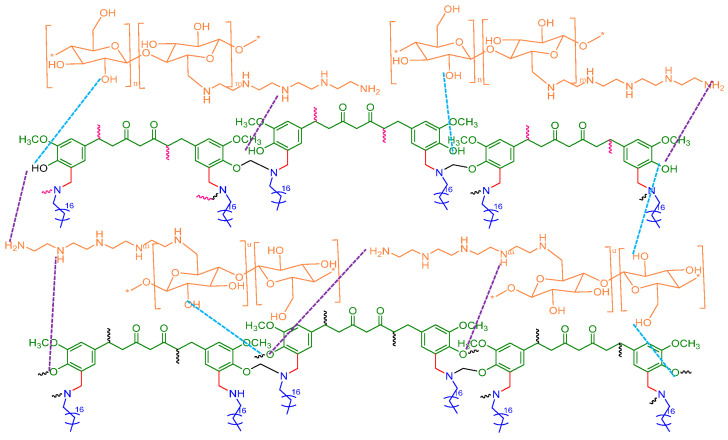
Polymerized structure of poly(C-st)/AC film.

**Figure 4:**
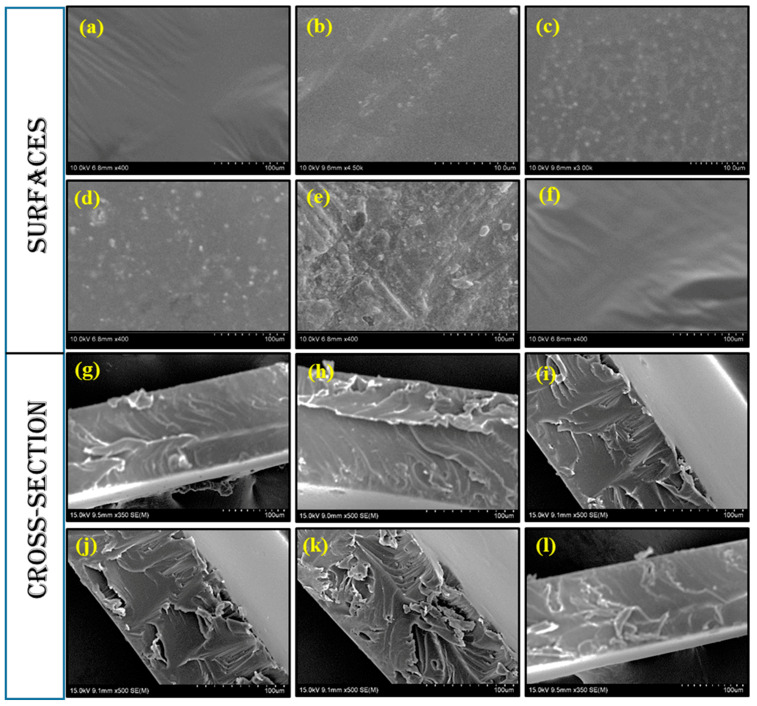
FE-SEM images of poly(C-st)/AC films: poly(C-st)/AC (100/0) (**a**,**g**); poly(C-st)/AC (80/20) (**b**,**h**); poly(C-st)/AC (60/40) (**c**,**i**); poly(C-st)/AC (40/60) (**d**,**j**); poly(C-st)/AC (20/80) (**e**,**k**); and poly(C-st)/AC (0/100) (**f**,**l**).

The top-view and cross-sectional surface morphologies of the poly(C-st) and poly(C-st)/AC films were examined by SEM micrographs and are displayed in Figure 4a,l. The top-view images show that both of the neat films [i.e., poly(C-st) and AC] have a smooth surface (Figure 4a,f), whereas, for the poly(C-st)/AC composite films (Figure 4b–e), the smooth surface was collapsed, and two different phases were observed with homogeneous distribution. This uniform distribution is mainly attributed to the formation of Mannich bridges, which allow molecules to be arranged in an orderly manner. The cross-section images of the poly(C-st)/AC films displayed in Figure 4g–l reveal that the microstructures are strongly compressed, smooth, and without visible pores, even with the increased AC content (Figure 4k). So, we can say that high temperatures are conducive to the cross-linking interactions between poly(C-st) and AC [45]. The ring-opening reaction, the formation of imine bonds, the H-bonding interactions, and the stacking of polybenzoxazine between the AC backbones resulted in a highly cross-linked network structure in the film as a result of these covalent interactions. The film is void of pores as it is highly cross-linked (Scheme 4).

### 3.5. Tensile Properties of Poly(C-st)/AC Films

**Figure 5:**
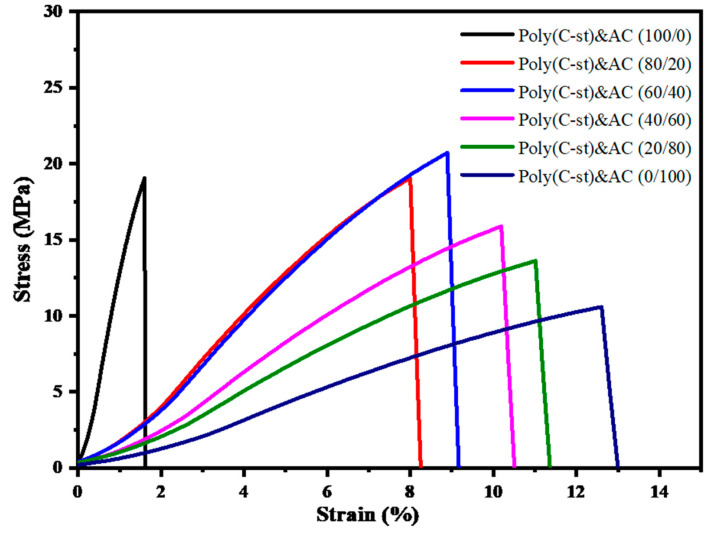
Stress–strain curves of poly(C-st)/AC films.

Figure 5 displays the stress/strain curves for the neat poly(C-st), neat AC, and poly(C-st)/AC films, and Table 3 summarizes the data obtained from them. There is a strong relationship between the average molecular weight of the neat polymers and their mechanical properties, which are strongly influenced by the fabrication conditions, including the amount of acid present in the aqueous solution. Tensile stress is the force exerted per unit of the cross-sectional area of the object, whereas the tensile strain is the extension per unit of the original length of the object. The neat polybenzoxazine, poly(C-st), can withstand a stress of about 18.21 MPa but has much less elongation at a break of 1.65%. This is obvious as Pbz is very brittle in nature, whereas, for the neat AC film, the reverse behavior is obtained, i.e., it can withstand stress of about 9.41 MPa but can elongate more with an elongation at a break value of 13.41%. The synergistic behavior could be clearly observed in all the poly(C-st)/AC films. Even with the incorporation of 20% AC into Pbz, its elongation at the break was drastically improved to 8.47%. Among the composite films, poly(C-st)/AC (60/40) shows the highest tensile stress value of about 22.07 MPa, with an improved elongation at a break value of 9.39%, whereas poly(C-st)/AC (20/80) has the highest elongation at the break value of 11.61%, with a tensile stress of 12.53 MPa. Polybenzoxazines with this type of hydrogen bonding interaction at the end of each chain have a lower elongation at the break, which is likely due to the presence of secondary amine groups and free phenolic moiety groups that act as additional cross-links [31]. This additional cross-linking site increases the cross-link density (CLD) of the network structure and thus contributes to the film stiffness. However, when AC is incorporated into the poly(C-st) network there is an introduction of flexible AC segments within the stiffened poly(C-st). This probably results in increased elongation at the break value of poly(C-st)/AC with increased AC content. The synergistic effect produced by the physicochemical cross-linking of AC with a second polymer on the mechanical properties of their films has been reported. The blending of AC with poly(C-st) polymer showed that the properties of the cross-linked films were mainly dependent on the degree of polymerization and the average molecular weight. The effect of varying the thermal treatment temperature on the tensile properties of the poly(C-st)/AC film was studied in detail for the poly(C-st)/AC (60/40) sample. The stress−strain curves of the poly(C-st)/AC (60/40) film after curing were studied with respect to their cross-linking. As the thermal treatment condition increases, both the tensile modulus and the tensile strength increase, whereas the elongation at the break decreases. This behavior is attributed to the increase in cross-link density coupled with the increased thermal treatment temperature, which leads to the further ring-opening polymerization of the benzoxazine structure and results in the formation of a more rigid three-dimensional network. The lower elongation at the break in this type of polybenzoxazine/AC film is presumably due to the hydrogen bonding of the free phenolic moieties and secondary amine groups that act as additional physical cross-links between the chains of the AC. As a result of the network structure, the cross-linking contributes stiffness to the films.

### 3.6. Thermal Stability of Poly(C-st) and Poly(C-st)/AC Films

**Figure 6:**
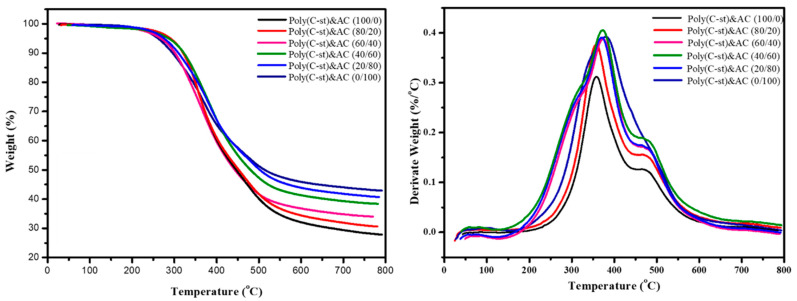
TGA thermograms of poly(C-st)/AC films.

The thermal stability test was conducted under a nitrogen atmosphere on the cured poly(C-st) and poly(C-st/AC) films; they are represented in Figure 6, and their data are tabulated in Table 4. The figure shows that for the neat poly(C-st), neat AC, and poly(C-st)/AC films, the maximum weight loss occurs between 230 and 500 °C, which is attributed to the degradation of the aliphatic content of the Pbz moiety and amino cellulose [46]. In addition, the poly(C-st) and poly(C-st/AC) cross-linked polymers function more consistently at temperatures between 250 and 350 °C. With the increasing AC content in the poly(C-st)/AC blend, there is a decrease in their degradation temperature. Furthermore, the poly(C-st/AC) films have a significantly higher thermal stability than the neat poly(C-st). The composite film, poly(C-st)/AC (60/40), has the highest thermal stability of 358 °C at 10% degradation. Therefore, it is clearly observed from the results that the thermal stability of the neat poly(C-st) film has been improved to a greater extent by making a composite film with AC. Moreover, the char yield at 800 °C was also increased from 28.2% [neat poly(C-st)] to 41.6% [poly(C-st)/AC (20/80)].

### 3.7. Flame Retardant Properties of *Poly(C-st) and Poly(C-st)/AC Films*

Based on their limiting oxygen index (LOI) values, the neat poly(C-st) and poly(C-st)/AC films were analyzed for flame retardancy. The LOI values are calculated using the van Krevelan and Hofytzer equation [47], based on the char yield (CY) values from the TGA analyses:

LOI = 17.5 + 0.4 (CY)(1)

In both the neat and the poly(C-st)/AC films, the LOI values exceed the threshold value of 26. Material with an LOI value higher than this threshold limit is generally considered self-extinguishing and flame retardant. Based on the obtained results, the synthesized poly(C-st) and poly(C-st)/AC films satisfy these properties. Hence, the thermal properties, including the degradation temperature, char yield values, and LOI values, are greatly improved for the poly(C-st)/AC composite films.

## 4. Conclusions

A simplified method for the fabrication of composite films from C-st and AC was adopted in this work. The structure of the synthesized benzoxazine monomer, C-st, has been confirmed by the FT-IR and ^1^H-NMR techniques, showing the oxazine ring vibration at 940 cm^−1^ and signals due to the oxazine ring protons at 4.6 and 5.4 ppm. Both the ring-opening mechanism of C-st and the cross-linking mechanism between C-st and AC pave the way for the synergistic effect to be pronounced in poly(C-st)/AC films through chemical and hydrogen bonds. Moreover, the incorporation of amino cellulose reduces the onset curing temperature of C-st from 163 to 152 °C, which further confirms that AC reacts with C-st and affects its polymerization process. The poly(C-st)/AC composite films with varying C-st contents were investigated, and it was found that the high C-st contents and elevated drying temperatures resulted in higher performances, playing a positive role in the improvement of the mechanical properties and thermal stability of composite films. It is interesting to note that the composite films have a higher tensile strength and modulus than the AC films [the tensile strength of poly(C-st)/AC (60/40) is 22.07 MPa]. As a result of these findings, composite films with enhanced thermal and mechanical properties may be able to enhance the functional applications of cellulose and possibly even open up opportunities for cellulose-based materials to be used as packing materials and refractory materials in industries. Furthermore, this approach has the potential to lead to outstanding growth for the polymeric industry in high-end fields. This way of blending synthetic polymer with natural polymer brings out their synergistic effects and provides superior mechanical and thermal properties in a wide range of applications, including packing materials, electronic motherboards, and membrane separations.

## Figures and Tables

**Table 1:** Weight ratios of C-st and AC.

Sample	Benzoxazine Monomer (C-st) (g)	Amino Cellulose (AC) (g)
(C-st) and AC (100/0)	5.0	0
(C-st) and AC (80/20)	4.5	0.5
(C-st) and AC (60/40)	4.0	1.0
(C-st) and AC (40/60)	3.5	1.5
(C-st) and AC (20/80)	3.0	2.0
(C-st) and AC (0/100)	0	5.0

**Table 2:** DSC data of C-st/AC blends.

Sample	T_onset_ (°C)	T_max_ (°C)	T_final_ (°C)	∆H (J/g)
(C-st) and AC (100/0)	163	175	184	228
(C-st) and AC (80/20)	160	176	188	205
(C-st) and AC (60/40)	158	177	192	161
(C-st) and AC (40/60)	155	181	195	93
(C-st) and AC (20/80)	152	183	198	63

**Table 3:** Data obtained from stress–strain curves.

Sample	Tensile Stress (MPa)	Tensile Strain at Break (%)
Poly(C-st) and AC (100/0)	18.21 ± 0.32	1.65 ± 0.32
Poly(C-st) and AC (80/20)	18.83 ± 0.53	8.47 ± 0.24
Poly(C-st) and AC (60/40)	22.07 ± 0.74	9.39 ± 0.71
Poly(C-st) and AC (40/60)	15.57 ± 0.83	10.36 ± 0.86
Poly(C-st) and AC (20/80)	12.53 ± 0.62	11.61 ± 0.53
Poly(C-st) and AC (0/100)	9.41 ± 0.12	13.41 ± 0.23

**Table 4:** Data obtained from TGA thermograms.

Sample	T_i_ (°C)	T_10_ (°C)	Char Yield (%)	LOI
Poly(C-st) and AC (100/0)	228	328	28.2	28.8
Poly(C-st) and AC (80/20)	224	336	32.3	30.4
Poly(C-st) and AC (60/40)	213	358	35.5	31.7
Poly(C-st) and AC (40/60)	197	344	39.8	33.4
Poly(C-st) and AC (20/80)	189	318	41.6	34.1
Poly(C-st) and AC (0/100)	176	316	43.3	34.8

## Data Availability

The data presented in this study are available on request from the corresponding author.
